# An Experimental Model of Vasovagal Syncope Induces Cerebral Hypoperfusion and Fainting-Like Behavior in Awake Rats

**DOI:** 10.1371/journal.pone.0163280

**Published:** 2016-09-22

**Authors:** Devin W. McBride, Cesar Reis, Ethan Frank, Damon W. Klebe, John H. Zhang, Richard Applegate, Jiping Tang

**Affiliations:** 1 Department of Physiology & Pharmacology, Loma Linda University, Loma Linda, California, United States of America; 2 Department of Anesthesiology, Loma Linda University, Loma Linda, California, United States of America; 3 Department of Neurosurgery, Loma Linda University, Loma Linda, California, United States of America; 4 Loma Linda University School of Medicine, Loma Linda, California, United States of America; University of PECS Medical School, HUNGARY

## Abstract

Vasovagal syncope, a contributing factor to elderly falls, is the transient loss of consciousness caused by decreased cerebral perfusion. Vasovagal syncope is characterized by hypotension, bradycardia, and reduced cerebral blood flow, resulting in fatigue, altered coordination, and fainting. The purpose of this study is to develop an animal model which is similar to human vasovagal syncope and establish an awake animal model of vasovagal syncope. Male Sprague-Dawley rats were subjected to sinusoidal galvanic vestibular stimulation (sGVS). Blood pressure, heart rate, and cerebral blood flow were monitored before, during, and post-stimulation. sGVS resulted in hypotension, bradycardia, and decreased cerebral blood flow. One cohort of animals was subjected to sGVS while freely moving. sGVS in awake animals produced vasovagal syncope-like symptoms, including fatigue and uncoordinated movements; two animals experienced spontaneous falling. Another cohort of animals was preconditioned with isoflurane for several days before being subjected to sGVS. Isoflurane preconditioning before sGVS did not prevent sGVS-induced hypotension or bradycardia, yet isoflurane preconditioning attenuated sGVS-induced cerebral blood flow reduction. The sGVS rat model mimics elements of human vasovagal syncope pathophysiology (hypotension, bradycardia, and decreased cerebral perfusion), including behavioral symptoms such as fatigue and altered balance. This study indicates that the sGVS rat model is similar to human vasovagal syncope and that therapies directed at preventing cerebral hypoperfusion may decrease syncopal episodes and reduce injuries from syncopal falls.

## Introduction

Vasovagal syncope (VVS) is characterized by a transient, self-limited loss of consciousness: loss of postural tone, collapse, and spontaneous recovery [1]. Between 20–50% of adults will experience at least one syncopal episode during their lifetime, and VVS is most common in the elderly with up to 23% of individuals over 70 years old affected and a 30% chance of reoccurrence [2]. Each year over 400,000 new syncope patients are diagnosed with VVS, of which 2–5% require emergency room visits, placing an annual burden of $2.4 billion on U.S. healthcare [3, 4].

The mechanism underlying VVS is not fully understood, yet the current thinking is decreased venous return to the heart causes vigorous contraction of the myocardium against inadequately filled atria, triggering the Bezold-Jarisch reflex resulting in paradoxical hypotension, bradycardia [1, 5], and consequent decreased cerebral perfusion, leading to a loss of consciousness [5]. The head-up tilt test is valuable in diagnosing VVS and understanding its mechanism. Head-up tilt tests on patients (mean age: 42.5 years) have shown that sympathetic nerve activity and myocardial contractility are reduced preceding the onset of syncope, followed by a lowering of blood pressure [6, 7].

In a series of compelling experiments, Cohen and colleagues proposed a rat model of VVS using sinusoidal galvanic vestibular stimulation (sGVS) [8–10]. Their groups found that sGVS could produce temporary hypotension and bradycardia. These studies imply that sGVS in rats mimics human vasovagal responses through activation of the otolith system [9]. While Cohen and colleagues have provided considerable research in understanding the role of sGVS on precipitating the vasovagal response, a critical aspect, and the final sequela, of VVS has not been explored: cerebral perfusion.

Despite VVS pathophysiology including the reductions in blood pressure and heart rate, VVS culminates in loss of conscious, typically leading to a fall [11]. Although the consensus is that cerebral hypoperfusion ultimately causes the fainting episode, currently there is limited research and understanding of the role cerebral blood flow (CBF) plays in VVS pathophysiology. To date, few clinical studies have been published measuring CBF before and during syncope. In one study by Grubb *et al*., thirty syncope patients were subjected to the head-up tilt test. During syncope, the mean CBF was observed to be 46 ± 17% lower than Baseline values [12]. A second study evaluated CBF changes in syncope patients with no bradycardia or hypotension during the episode. In these patients, the mean CBF was still markedly reduced during syncope (26 ± 13% lower than Baseline values) [13]. Sung *et al*. also investigated CBF changes during syncope in patients and found that standing or head-up tilt test induced syncopal-related lowering of the mean CBF by 32 ± 11.6% (standing) and 30 ± 16.7% (head-up tilt test) [14]. These studies, as well as the VVS paradigm, consider cerebral hypoperfusion as the primary sequelae of VVS. Therefore, an animal model of VVS must show reduction in cerebral blood flow during syncope. Additionally, VVS patients are plagued by spontaneous loss of consciousness. Therefore, a second key to an animal model of VVS is that behavioral changes mimicking VVS symptoms are experienced by awake animals.

Herein we utilize the sGVS model in rats to examine its impact on CBF and, in awake animals, behavior changes caused by sGVS. Our first hypothesis is sGVS in rats results in decreased blood pressure and heart rate (HR), followed by reduced CBF, mimicking vasovagal response in humans. Our second hypothesis is sGVS in awake animals will induce VVS-like symptoms, namely fatigue followed by spontaneous fainting and recovery. Our third hypothesis is isoflurane preconditioning will induce cardio- and neuro-protective mechanisms, thereby attenuating the effects of sGVS on blood pressure, HR, and CBF.

## Materials and Methods

All experiments were carried out in accordance to the methods and procedures approved by the Loma Linda University IACUC and Research Protection Programs, and conducted in compliance with the *NIH Guidelines for the Use of Animals in Neuroscience Research*. A protocol was in place for early termination of experiments if animals became severely ill (determined by the Loma Linda University veterinary staff).

Adult male Sprague-Dawley rats (280–320 g) were obtained from Harlan Laboratories (Harlan Laboratories, Indianapolis, IN, USA). Animals were housed in a humidity and temperature controlled room on a 12 hour light-dark cycle, and given food and water *ad libitum*. During all surgical procedures and methods during which rats were anesthetized, the body temperature was maintained at 37 ± 0.5°C using a heating pad controlled using a rectal probe. Animals were continuously monitored during all surgical procedures and methods. Animals were randomly assigned to the groups in each experiment. After surgical procedures, betadine was applied to the wound(s), and buprenorphine was administered subcutaneously (0.01 mg/kg). At the end of the experiment, rats were euthanized via decapitation after deeply anesthetized with 5% isoflurane. No animals died before the experimental endpoints were achieved.

Sixty-six rats were used in this study. Four animals were used in a pre-experiment to determine the optimal stimulation parameters for lowering mean arterial pressure (MAP) and HR, while depressing CBF. Experiment 1 investigated the effect of sGVS on CBF (groups: Sham and sGVS, n = 10/group). Experiment 2 examined the effect of sGVS in awake, freely moving rats (groups: Sham and sGVS, n = 6/group). Experiment 3 evaluated isoflurane preconditioning as a potential therapy to prevent sGVS sequelae (groups: Sham, sGVS, and sGVS + isoflurane preconditioning, n = 10/group). Sham animals were normal rats which underwent all surgeries and electrode placement, had the MAP, HR, and CBF measured, but without ever undergoing sGVS stimulation. Additional methodological details are available in S1 File.

### sGVS

sGVS was induced using a computer-controlled stimulator (Grass Technologies, West Warwick, RI, USA) which generates sinusoidal currents and applied via electrodes [8–10]. After laser Doppler probe placement, two Ag/AgCl needle electrodes were inserted into the skin over the mastoids. The computer-controlled stimulator generated sinusoidal currents binaurally (2–4 mA, 0.025–0.5 Hz). Rats were kept under anesthesia for a total of 67 minutes: 30 minutes for surgeries (femoral artery catheterization, laser Doppler probe placement, and electrode placement), during measurement of the baseline (for MAP, HR, and CBF, 4 minutes), during sGVS stimulation (3 minutes), and for 30 minutes post-stimulation.

### Pre-Experiment: Determine the Optimal Parameters for Inducing sGVS

In four rats, to determine the optimal stimulation parameters for inducing MAP and HR reductions, various combinations of amplitude and frequencies were tested: 2 or 4 mA at 0.025–0.5 Hz. The optimal stimulation parameters were determined to be the frequency and amplitude which induced the greatest, most reproducible drop in MAP and HR among each of the four animals. CBF was monitored in each rat to identify any CBF changes during stimulation using the various sets of stimulation parameters. These four animals were not used in any other aspect of this study. Rats were deeply anesthetized with 5% isoflurane and then decapitated after completing the experiment.

### Experiment 1: Effect of sGVS on Cerebral Blood Flow

Twenty animals were randomly assigned to either the Sham or sGVS group (n = 10/group). Sham animals were normal rats which underwent all surgeries (burr hole, femoral artery catheterization) and electrode placement, had the MAP, HR, and CBF measured, but without ever undergoing sGVS stimulation. These animals were anesthetized with isoflurane for the same duration as animals subjected to sGVS and were monitored for MAP, HR, and CBF changes for the same duration as sGVS animals (4 minutes of “Baseline”, 3 minutes of “Stimulation” without electrical stimulation, and 30 minutes “Post-Stimulation”). Sham animals were used as the control group.

One day before sGVS, rats were anesthetized and a burr hole was drilled in the skull (center: 5 mm proximal to the coronal suture, 4 mm right lateral of the sagittal suture) using a microdrill. Then bone wax was applied and the skin sutured. Betadine was applied to the wound and buprenorphine was administered subcutaneously (0.01 mg/kg). The animal was allowed to recover before being returned to its home cage.

On the day of sGVS, a PE50 catheter was inserted and advanced 10–15 mm into the femoral artery of an anesthetized rat. The catheter was connected to a transducer for continuous measurement of MAP and HR using a blood pressure analyzer (Digi-Med BPA 400a, Micro-Med, INC., Louisville, KY, USA) and the DMSI-400 software (Micro-Med, INC., Louisville, KY, USA). After femoral artery catheterization, the burr hole was reopened, exposing the brain tissue. A laser Doppler probe (OxyFlo probe, MNP100XP, AdInstruments Inc., Colorado Springs, CO, USA) was used for continuous measurement of CBF using a blood flow monitor (INI191, AdInstruments Inc., Colorado Springs, CO, USA) and PowerLab (PL3504, AdInstruments Inc., Colorado Springs, CO, USA) with the LabChart software (LabChart Pro, AdInstruments Inc., Colorado Springs, CO, USA).

sGVS was induced for three minutes with a 4 mA amplitude and 0.025 Hz frequency. MAP, HR, and CBF were continuously monitored for four minutes before sGVS, during stimulation (3 minutes), and for 30 minutes post-sGVS. Thirty minutes after stimulation, the laser Doppler probe was removed and the rats were deeply anesthetized with 5% isoflurane and then decapitated.

### Experiment 2: Effect of sGVS on Awake, Freely Moving Animals

Twelve animals were used to examine the effects of sGVS on awake animals. Animals were randomly assigned to either the Sham or sGVS group (n = 6/group). Sham animals had electrodes placed, but received no stimulation. MAP, HR, and CBF were not monitored in any animal for experiment 2. The only surgical procedure performed was electrode placement.

Electrodes were placed while the animal was under anesthesia. Total duration of isoflurane exposure during electrode placement was not longer than 10 minutes (range: 6–10 minutes). Animals were allowed to recover from the effects of isoflurane for a minimum of 50 minutes (range: 50–70 minutes) in a cage identical to its home cage without bedding, food, and water. After the animal fully recovered, sGVS was induced for 3 minutes. The animal’s behavior was recorded for 5 minutes before stimulation, during stimulation (3 minutes) and for 60 minutes post-stimulation. Immediately after recording stopped (60 minutes post-stimulation), animals were deeply anesthetized and decapitated.

### Experiment 3: Effect of Isoflurane Preconditioning on sGVS

Thirty animals were randomly assigned to either the Sham, sGVS, or isoflurane preconditioning followed by sGVS group (n = 10/group). Sham animals served as the control group and underwent all procedures as described in Experiment 1. All animals receiving sGVS (sGVS only group and isoflurane preconditioning followed by sGVS group) underwent all surgical procedures as described in Experiment 1.

Ten animals were subjected to five days of isoflurane preconditioning. Daily, isoflurane (2.5%) was administered to animals for 90 minutes. The isoflurane preconditioning regimen was completed five days before subjecting rats to sGVS. Four days after completing the isoflurane preconditioning regimen, rats underwent the surgical procedure for creating the burr hole (see Experiment 1 and S1 File for details). One day later (five days after completing the isoflurane preconditioning regimen), rats were subjected to femoral artery catheterization, laser Doppler probe placement, and sGVS as described in Experiment 1. MAP, HR, and CBF were monitored before, during, and for 30 minutes post-stimulation as described in Experiment 1. Animals were anesthetized before, during, and after stimulation. All animals were deeply anesthetized and decapitated 30 minutes after stimulation (immediately after recording was completed).

### Data Processing and Statistical Analysis

All raw data was processed and analyzed by a blinded investigator. The data was divided into three sections: Baseline (minutes 0–4), Stimulation (minutes 3–7), and Post-Stimulation (minutes 7–37). Within each section the data was averaged and the standard deviation (SD) calculated. Upon processing, the Post-Stimulation data had very large SD due to the variation in the return to baseline values, thus the Post-Stimulation section was subdivided into 0–5 Min, 5–10 Min, 10–20 Min, and 20–30 Min Post-Stimulation. Since there was significant variation between animals (within the same group) in the raw Baseline values for the physiological parameters measured, the change from Baseline values were computed for each section/subsection. The data is presented as the mean (% change from Baseline) ± SD. In Experiment 2, the behavioral changes for animals during and post-sGVS are anecdotally described since functional tests specifically related to VVS symptoms were lacking. Finally, the data was processed to determine the amount of time after beginning stimulation that was required to observe a rapid, sustained drop in CBF (CBF drop delay). The local minimums for the data obtained during stimulation were identified. Each local minimum was examined for 1) being greater than 3 SD change from Baseline, and 2) sustained depression compared to Baseline (more than 15 seconds). The delay in CBF drop was the amount of time after beginning stimulation that the minimum occurred. Data was analyzed using two-way ANOVA with Sidak’s *post-hoc* test (GraphPad Prism 6, La Jolla, CA). p<0.05 was considered statistically significant; p<0.1 was considered as a tendency.

## Results

**Fig 1:**
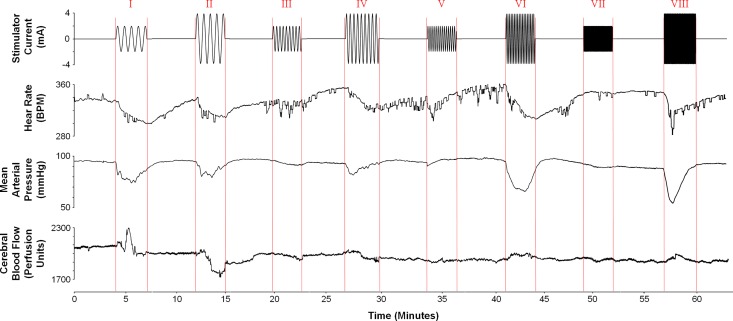
Effects of Various Sets of Stimulation Parameters on HR, MAP, and CBF. A representative plot of the stimulator current (mA), heart rate (BPM), mean arterial pressure (mmHg), and cerebral blood flow (perfusion units) is shown. Baseline data was collected for 4 minutes before beginning stimulation (minutes 0–4). Five minutes of recovery was allowed after each stimulation event. Stimulation occurred for 3 minutes using eight different sets of stimulation parameters: 2 mA at 0.025 Hz (I), 4 mA at 0.025 Hz (II), 2 mA at 0.05 Hz (III), 4 mA at 0.05 Hz (IV), 2 mA at 0.1 Hz (V), 4 mA at 0.1 Hz (VI), 2 mA at 0.5 Hz (VII), and 4 mA at 0.5 Hz (VIII). Red lines highlight the start and stop of each stimulation event. The greatest drop in CBF (13.9%) occurred for the 4 mA, 0.025 Hz current (II).

In the pre-experiment, four rats were used to determine the optimal stimulation parameters required to induce reproducible vasovagal-like response: reduction in blood pressure and heart rate. Each animal was subjected to sGVS using the following stimulation parameters: current amplitude was either 2 or 4 mA, frequencies were 0.025, 0.05, 0.1, or 0.5 Hz. The optimal stimulation parameters for inducing cardiovascular depression in rats was found to be a 4 mA current at 0.025 Hz (Fig 1II). This set of stimulation parameters induced depression of MAP and HR in each animal. Monitoring of the cerebral blood flow indicated that this set of stimulation parameters was also capable for causing a reduction in cerebral blood flow, as shown by decreased cerebral perfusion units measured using laser Doppler flow. Other sets of parameters were observed to produce MAP and HR depression in some animals but not others. For example, the representative plot (Fig 1) shows that the 4 mA at 0.05 Hz, 4 mA at 0.1 Hz and 4 mA at 0.5 Hz currents also induced marked decreases in MAP and HR (IV, VI, and VIII, respectively). However, these sets of stimulation parameters did not cause reductions in MAP and HR in some animals. Thus, these sets of stimulation parameters were unable to cause reproducible lowering of MAP and HR. Additionally, the 4 mA at 0.1 Hz and 4 mA at 0.5 Hz currents did not have any consequent CBF reductions (VI and VIII, respectively) despite the marked lowering of MAP and HR. Based on the preliminary experiment, a 4 mA current at a frequency of 0.025 Hz was used in Experiments 1–3.

No mortality was observed in this study. A total of seven animals were removed from the study since no vasovagal-like response was observed (*i*.*e*. lack of a decrease in MAP or HR combined by a lack of oscillatory response of MAP or HR): two from the sGVS group in Experiment 1, two from the sGVS group in Experiment 3, and three from the sGVS + Isoflurane group in Experiment 3. In total, data was collected and analyzed for 18 animals in Experiment 1 (n = 10 for Sham, n = 8 for sGVS), 12 animals in Experiment 2 (n = 6 for Sham, n = 6 for sGVS), and 25 animals in Experiment 3 (n = 10 for Sham, n = 8 for sGVS only, and n = 7 for sGVS + Isoflurane Preconditioning).

### Experiment 1 –sGVS Causes Decreased Cerebral Blood Flow

**Fig 2:**
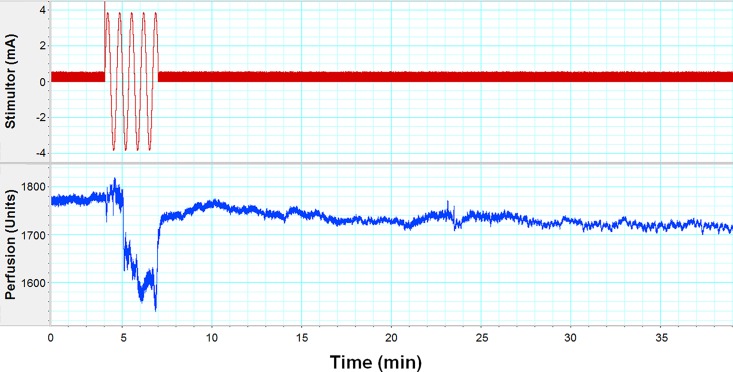
sGVS Causes CBF Reduction. Representative plot of CBF (bottom, blue plot) changes during sGVS (top, red plot). Baseline CBF (~1780 perfusion units) was collected for 4 minutes before beginning sGVS (at minute 4). Approximately 1 minute after starting sGVS a significant drop in CBF was observed, which was maintained throughout stimulation. After sGVS, CBF recovers, but does not return to baseline values.

**Fig 3:**
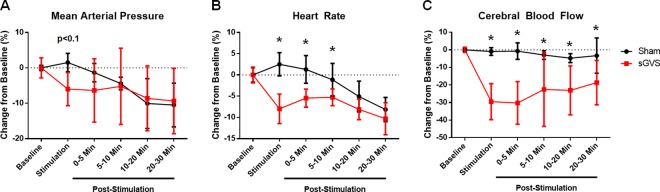
MAP, HR, and CBF Changes Induced by sGVS. **(A)** MAP tends to decrease during sGVS (p<0.1). After sGVS, MAP recovers. Prolonged anesthesia causes a steady MAP drop in all rats (Sham: p<0.05 for Baseline vs 10–20 and 20–30 Min Post-Stimulation, p<0.1 for 0–5 Min Post-Stimulation vs 20–30 Min Post-Stimulation; sGVS: p<0.1 between Baseline and 20–30 Min Post-Stimulation). **(B)** HR is significantly reduced for sGVS rats compared to Sham during sGVS (p<0.05), and for up to 10 Min Post-Stimulation (p<0.05). Prolonged anesthesia causes steady decline of HR in all rats (Sham: p<0.05 for Baseline vs 10–20 and 20–30 Min Post-Stimulation, p<0.05 for 0–5 and 5–10 Min Post-Stimulation vs 10–20 and 20–30 Min Post-Stimulation; sGVS: p<0.05 for Baseline vs each time range post-sGVS, p<0.05 for 0–5 and 5–10 Min Post-Stimulation vs 20–30 Min Post-Stimulation). **(C)** CBF is significantly reduced during sGVS compared to Sham (p<0.05) and remains depressed for up to 30 minutes post-sGVS (p<0.05). Prolonged anesthesia does not significantly lower CBF in either sham or sGVS rats. * p<0.05 for Sham vs sGVS for the given time variable.

sGVS in rats causes a delayed but significant drop in CBF (Fig 2). Compared to sham animals, sGVS causes a decrease in HR (p<0.05) and CBF (p<0.05), and a tendency to decrease mean arterial pressure (MAP) (p<0.1) (Fig 3). Upon halting sGVS, MAP rapidly returned to a level indistinguishable from that of Sham (p>0.1). However, HR remained depressed compared to that of Sham for 10 minutes post-sGVS (p<0.05 for 0–5 and 5–10 Min Post-Stimulation). CBF was significantly lowered compared to that of Sham up to 30 minutes post-sGVS (p<0.05 for each time range).

A tendency to decrease over time was observed in Sham and sGVS rats for MAP (Sham: p<0.05 for Baseline vs 10–20 and 20–30 Min Post-Stimulation; sGVS: p<0.1 for Baseline vs 10–20 and 20–30 Min Post-Stimulation) and HR (Sham: p<0.05 for Baseline vs 10–20 and 20–30 Min Post-Stimulation; sGVS: p<0.05 for Baseline vs 0–5, 10–20, and 20–30 Min Post-Stimulation). No time-dependent changes in CBF were observed for Sham. Other than the marked reduction in CBF caused by sGVS, the CBF in rats subjected to sGVS was also unaffected over time.

### Experiment 2 –sGVS in Awake Animals Mimics Vasovagal Syncope Symptoms

**Fig 4:**
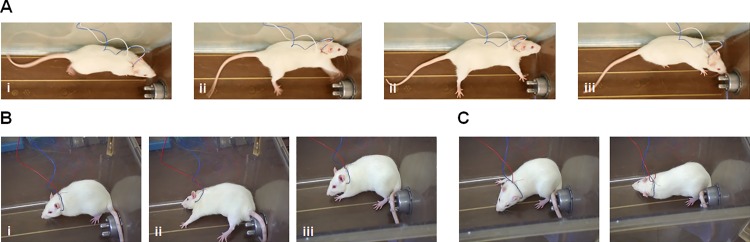
Behavioral Changes during sGVS in Awake Rats. Representative images of the changes in behavior observed during sGVS in awake rats. Sinusoidal galvanic vestibular stimulation in the awake animal induces similar symptoms as that experienced by VVS patients. Awake animals during stimulation exhibit signs of fatigue-like behavior (reduced responsiveness and lethargy), labored breathing, altered coordination, and even faint-like behavior (*i*.*e*. falling). Representative images from fainting-like behavior in two animals are shown (**A**, **B**) with pre-faint-like stance (**i**), stance during the faint-like behavior (**ii**), and spontaneous recovery within 1–2 seconds after the onset of the faint-like behavior (**iii**). **(C)** Representative images of altered coordination and head swaying.

Awake, freely moving animals were subjected to sGVS to evaluate the effect of sGVS on behavior. Before beginning sGVS, all animals exhibited normal behavior (balance, movements, walking, body tone). Sham animals continued to have normal behavior throughout the duration of electrode implantation (1.5–2 hrs). At the onset of sGVS, rats’ behaviors began to change (Fig 4). First, the breathing rate began to fluctuate (slow and deep or shallow). Within 30 seconds, animals exhibited fatigue-like behavior, such as labored breathing and reduced responsiveness to external stimuli (sound and light). Between 0.5 and 2 minutes (and for the remainder duration of stimulation), rats began to show signs of abnormal movement; use of limbs was slower, head swaying and/or head rotation, body swaying and/or leaning towards one side. During this time, when animals walked, it was unbalanced/uncoordinated. During sGVS, 2 animals had spastic movements and 2 animals had fainting-like behavior. The latter two rats fell on their side, then had 1–2 seconds of either no movement or one limb spasm, then recovered spontaneously. After stopping stimulation, the animals subjected to sGVS exhibited fatigue-like behavior for up to 45 minutes after sGVS. Animals subjected to sGVS recovered normal behavior 28 ± 9.5 minutes post-stimulation (range: 15–45 minutes).

### Experiment 3 –Isoflurane Preconditioning Reduces the Effect of sGVS Cerebral Blood Flow Depression

#### Mean Arterial Pressure

**Fig 5:**
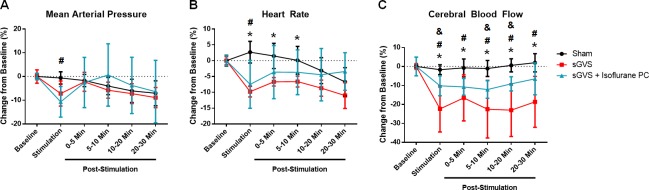
Effects of Isoflurane Preconditioning on MAP, HR, and CBF Changes During and Post-sGVS. **(A)** MAP is significantly reduced by sGVS in unconditioned and isoflurane preconditioned rats subjected to sGVS compared to Sham (p<0.1 Sham vs sGVS, p<0.05 Sham vs sGVS + Isoflurane PC). MAP recovers immediately upon stopping sGVS, but prolonged anesthesia has a tendency to steadily decrease MAP (Sham: p<0.1 Baseline vs 20–30 Min Post-S-Stimulation; sGVS: p<0.1 Baseline vs 20–30 Min Post-Stimulation). **(B)** During sGVS, HR is significantly lowered by sGVS in unconditioned and isoflurane preconditioned rats compared to Sham (p<0.05 for Sham vs sGVS and sGVS + Isoflurane PC). HR in unconditioned animals remains decreased for up to 10 minutes post-sGVS, while the HR of isoflurane preconditioned rats recovers after sGVS stops. Prolonged anesthesia causes continued decreased in HR for sham and unconditioned sGVS animals (Sham: p<0.1 between Baseline and 20–30 Min Post-Stimulation; sGVS: p<0.05 for Baseline vs 10–20 and 20–30 Min Post-Stimulation). **(C)** CBF is reduced by sGVS in unconditioned and isoflurane preconditioned animals compared to Sham (p<0.05 for Sham vs sGVS and sGVS + Isoflurane PC), yet isoflurane preconditioning attenuates the CBF reduction caused by sGVS (p<0.05 sGVS vs sGVS + Isoflurane PC). The CBF reduction caused by sGVS remains for up to 30 minutes post-stimulation (p<0.05 for Sham vs sGVS and sGVS + Isoflurane PC for each time frame Post-Stimulation). * p<0.05 for Sham vs sGVS for the given time variable, **#** p<0.05 for Sham vs sGVS + Isoflurane PC, **&** p<0.05 for sGVS vs sGVS + Isoflurane PC.

Compared to sham animals, sGVS decreased MAP in rats preconditioned with isoflurane (sGVS + Isoflurane Preconditioning (PC)) (p<0.05) and tended to decrease MAP in unconditioned animals (p<0.1) (Fig 5A). During sGVS, no difference was observed for MAP between unconditioned and isoflurane preconditioned rats. After stopping sGVS, the MAP of animals subjected to sGVS only and those that were isoflurane preconditioned returned to levels indistinguishable from that of Sham. MAP for Sham and sGVS only animals tended to decrease over time (p<0.1 Baseline vs 20–30 Min Post-Stimulation), which was not observed Post-sGVS in isoflurane preconditioned rats.

#### Heart Rate

Rats subjected to sGVS had reduced HR during stimulation compared to that of Sham for unconditioned and isoflurane preconditioned rats (p<0.05 for Sham vs sGVS and sGVS + Isoflurane PC) (Fig 5B). Isoflurane preconditioning did not attenuate the impact of sGVS on HR. The HR of animals subjected to only sGVS remained depressed for up to 10 minutes post-sGVS whereas HR of sGVS animals preconditioned with isoflurane returned to values that were not statistically different from that of Sham immediately after stopping stimulation (p<0.05 Sham vs sGVS at 0–5 and 5–10 Min Post-Stimulation). Over time, the HR in Sham animals and unconditioned animals subjected to sGVS decreased (Sham: p<0.05 Baseline vs 20–30 Min Post-Stimulation; sGVS: p<0.1 for Baseline vs 0–5 and 5–10 Min Post-Stimulation, p<0.05 for Baseline vs 10–20 and 20–30 Min Post-Stimulation), whereas isoflurane preconditioned sGVS rats’ HR did not have any tendency to decrease over time.

#### Cerebral Blood Flow

During stimulation, CBF is significantly reduced in animals subjected to sGVS compared to that of sham animals (p<0.05 Sham vs sGVS), and this reduction is sustained for up to 30 minutes post-stimulation (p<0.05 Sham vs sGVS for each time range post-sGVS) (Fig 5C). CBF of isoflurane preconditioned animals was also statistically lower than that of sham animals during stimulation (p<0.05 Sham vs sGVS + Isoflurane PC). In isoflurane preconditioned animals, the reduced CBF continued for up to 30 minutes after sGVS (p<0.05 Sham vs sGVS + Isoflurane PC for 0–5, 5–10, 10–20, and 20–30 Min Post-Stimulation). However, isoflurane preconditioned rats had a significantly attenuated CBF drop compared to unconditioned rats both during stimulation (p<0.05 sGVS vs sGVS + Isoflurane PC) and at each time range post-stimulation (p<0.05 sGVS vs sGVS + Isoflurane PC at 0–5, 5–10, 10–20, and 20–30 Min Post-Stimulation).

## Discussion

Sinusoidal galvanic vestibular stimulation in rats provides an experimental mimetic of human vasovagal-like response [8–10]. Herein, sGVS induced cardiovascular and cerebrovascular depression in rats, as well as resulting in VVS-like symptoms in awake animals. The reduction in CBF during sGVS was partially attenuated with isoflurane preconditioning despite isoflurane preconditioning having no effect on sGVS-induced lowering of MAP or HR.

The sGVS model has been previously shown to cause lower MAP and HR in rats through activation of the vestibular neurons in the otolith organs [9, 15]. In the landmark experiments by Cohen and colleagues, sGVS in rats was established as an experimental model mimicking human vasovagal response and VVS [8–10]. In that series of experiments, sGVS was found to cause rapid lowering of MAP and HR at stimulus amplitudes of 2–4 mA and frequencies of 0.008–0.4 Hz [8, 9] with the most effective stimulation frequency being 0.025 Hz [10]. Herein, our findings support the findings that the optimal stimulation parameters for inducing vasovagal-like responses in rats is a 4 mA current at 0.025 Hz. Additionally, we found that not only is sGVS capable of producing the cardio-vascular depression that mimics human VVS, but sGVS also results in marked reduction of CBF. While HR and MAP may contribute to the onset of VVS-induced fainting, VVS pathophysiology culminates in decreased cerebral perfusion (*i*.*e*. results in loss of consciousness) [5, 16].

### Similarities between the sGVS Rodent Model and Vasovagal Syncope in Patients

#### VVS Phase 1: Progressive Early Hypotension

In VVS patients, there are four distinct phases of vasovagal syncope: progressive early hypotension, terminal vasodilatation, the syncopal episode, and the postfaint phase. The first phase, termed “progressive early hypotension,” occurs for patients, during head-up tilt test, who are destined to develop syncope [5]. This phase is characterized by a slow but continuous lowering of blood pressure and cardiac output, as well as an increase in HR [5]. This phase may last up to 5 minutes. In rats, 7 of 16 animals subjected to sGVS showed a brief (less than 20 seconds) rise in HR, with only 3 of those 7 animals showing a slow decline in BP during that period. Four of the 7 animals which showed a rise in HR immediately following the start of stimulation had rapid lowering of blood pressure. Further studies need to be performed to determine if these observations in fact correlate with human VVS by measuring cardiac output [5].

#### VVS Phase 2: Terminal Vasodilatation

The second phase of VVS, termed “terminal vasodilatation,” occurs during the last 2 minutes before syncope [5]. The characteristics of this phase include a rapid reduction in blood pressure and a slowing of HR, with the decrease in HR occurring after the rapid drop in blood pressure [5]. While in humans the sequence of physiological changes leading to syncope may be induced by a number of factors [5, 17], sGVS in rodents more closely mimics this phase (typically skipping the progressive early hypotension phase). In the majority of animals, MAP rapidly decreases after stimulation is started [8–10], followed by slow decline in HR. A few animals (4 of 16) had a sharp decline in HR immediately following the blood pressure drop.

Also observed in humans during the second phase of VVS is a slow decline in CBF which precedes a rapid drop immediately before the syncopal episode [5]. In 11 of 16 rats, CBF did not significantly change from Baseline before the sharp drop occurred. Only 5 of 16 animals experienced a slow gradual decline in CBF preceding a rapid drop. In these five animals, the decline in CBF (before the drop-off) occurred for 21 ± 5.6 seconds, with one exception in which the gradual decrease in CBF happened over 120 seconds before the rapid drop.

#### VVS Phase 3: Syncope

The third phase of VVS is the transient loss of consciousness. In this phase, blood pressure and HR are at their minimum values and have experienced sharp falls just prior to the faint. However, this phase is primarily characterized by the rapid drop in CBF. In VVS patients, the rapid decrease in blood pressure and HR precedes the syncopal episode by 30–120 seconds, but can be variable [5, 18–23]. In the rat model of sGVS, a delay from the start of stimulation (and thus MAP and HR reductions) until the rapid drop in CBF was observed. Rats subjected to sGVS experienced a sharp reduction in CBF 51 ± 21.7 seconds after starting stimulation. When rats were preconditioned with isoflurane before sGVS, the delay in the CBF drop was 72 ± 36.4 seconds. If one assumes that the rapid drop in CBF in rats corresponds to the syncopal episode (*i*.*e*. loss of consciousness), then the delay in this drop is similar to the amount of time observed in human VVS [5, 18–23]. Interestingly, this delay in CBF drop observed in the sGVS rat model mirrors the “pre-syncope” window reported in humans. Therefore, this model may be utilized for examining therapies, administered during the pre-syncopal event, which are aimed at preventing the loss of consciousness.

To date, there has been a limited number of studies in which CBF was monitored during syncope. Grubb *et al*. performed two independent studies measuring the change in CBF in humans with VVS. In the authors’ first study, the mean CBF during syncope in twenty VVS patients dropped by 46 ± 17% [12]. The second study by Grubb *et al*. evaluated the changes in CBF during syncope in five patients in which hypotension and bradycardia was absent. Despite these patients not having reduced blood pressure or HR, they experienced a syncopal episode which correlated with a decrease in the mean CBF of 26 ± 13% during syncope [13]. Sung *et al*. observed reductions of the mean CBF in syncope patients during standing or head-up tilt test to be 32 ± 11.6% and 30 ± 16.7%, respectively [14]. In rats, sGVS induces changes in CBF of 22 ± 11.7% (S1 Fig). The minor differences in the percent CBF reduction during syncope between the rat sGVS model and human VVS may be attributed to the use of anesthesia in rats (*i*.*e*. isoflurane blocks some of the CBF depressive effects of sGVS).

Finally, sGVS in awake animals produces behavior similar to that observed in humans during VVS. During sGVS, awake animals were fatigued, had variable breathing, had uncoordinated movements, and had head swaying [19]. Two animals even had fainting-like behavior: spontaneous fall followed by 1–2 seconds of either no movement or jerky movements of one limb, then spontaneous recovery. Humans have been reported to experience a number of behavioral changes preceding the syncopal episode, including light-headedness, fatigue, blurred vision, sweating, shortness of breath, palpitations, and nausea [11, 19]. These symptoms are reported in the majority of VVS patients, with fatigue being the most common (in 92% of patients) [11].

#### VVS Phase 4: Postfaint Phase

The final phase of VVS is the postfaint phase which is characterized by recovery from the loss of consciousness. During this phase, blood pressure and HR recover immediately in the majority of VVS patients, however some patients remain hypotensive for up to 5 minutes [5]. In the sGVS rat model, MAP tends to return to levels indistinguishable from sham animals. However, due to the hypotensive effects of isoflurane, the MAP of sham animals begins to reduce Post-Stimulation. Yet the HR in sGVS rats remains depressed for up to 10 minutes after stimulation is stopped.

Another postfaint observation, is that most VVS patients (up to 75%) experience post-syncope behavioral changes, such as fatigue and lethargy, light-headedness, disorientation, nausea, confusion, palpitations, and altered mental status [11, 19]. Although the exact cause of these symptoms after VVS is unknown, the CBF in the rodent model of sGVS suggests that it may be a prolonged depression of CBF. Despite MAP and HR recovering within 15 minutes after stimulation in the rat model, CBF was reduced for up to 30 minutes after sGVS. This observation may provide a physiological explanation for the postfaint symptoms observed in the awake rat model (such as extreme fatigue) as well as the postfaint symptoms reported by VVS patients [11, 19].

### Limitations and Future Studies

To date, there still remains some aspects of VVS which need to be investigated in the sGVS rat model. First, the mechanism of sGVS is still unclear. VVS in humans is related to vagal activation and sympathetic inhibition. Since the mechanism of sGVS is not known, it cannot yet be labeled as a true model of VVS. Second, the mechanism of human VVS is characterized by decreased cardiac output (lowered HR and/or stroke volume) resulting in hypotension, culminating in cerebral hypoperfusion [16]. The current understanding of the sGVS rat model does not focus on cardiac output, but rather HR. Future studies should investigate cardiac output during sGVS in rats. Third, the decreased blood pressure is within the range of cerebral autoregulation (50–150 mmHg), thus healthy humans are unlikely to experience reduced CBF. However, studies on the blood pressure and CBF changes in VVS patients have also found that syncope can be induced despite the reduced blood pressure being within the cerebral autoregulation range [7, 12, 14]. These reports are also supported by Grubb *et al*. and Sung *et al*. in studies which identified significant changes in CBF during syncope in patients with reduced blood pressure that is within the cerebral autoregulation range [12, 14]. Furthermore, in syncope patients, CBF lowering and syncope can occur even in the absence of hypotension and bradycardia [13]. Thus syncope may rely less on blood pressures outside of the cerebral autoregulation range than previously thought. However, this phenomenon, as well as the effects of sGVS on CBF need to be examined in greater detail to understand the cause of VVS despite blood pressure being within the cerebral autoregulatory range. Fourth, the mean drop in blood pressure induced by sGVS in rats is 8% which is less than observed in syncopal patients subjected to head-up tilt testing (approximately 20–30% drop in blood pressure) [5, 12]. The mechanism causing the CBF reduction in rats with only an 8% drop in blood pressure remains to be investigated, but may in part be due to the animals being anesthetized, thereby having a lower baseline MAP (compared to the MAP in awake rats), whereas VVS patients are fully awake when syncope occurs. Fifth, the results of Experiment 2 (observing sGVS in freely moving rats) are qualitative. Currently no behavioral tests are available for quantifying the altered behavior of rats during sGVS. In future studies we will develop a behavioral test(s) for identifying behavioral changes in rodents during sGVS.

## Conclusion

Herein, we advance the understanding of the sGVS rat model and its use as a pre-clinical mimetic of VVS, as well as investigate a therapy for vasovagal syncope. To our knowledge, we are the first to show that sGVS causes a CBF decrease, in addition to MAP and HR, with a rapid decrease in MAP and HR preceding CBF reduction. After stimulation, while MAP and HR recover, CBF remains depressed for up to 30 minutes post-sGVS. We are also the first to show that sGVS in awake rats produces VVS-like symptoms, including fatigue and fainting-like behavior. Finally, we are the first to begin investigating therapies for VVS using the sGVS rat model with the focus on attenuating cerebral hypoperfusion. We identified that isoflurane preconditioning reduces CBF depression caused by sGVS, which may point to potential therapies that can decrease syncopal episodes. The current study not only provides the basis for using the sGVS rat model to examine the vasovagal response and develop novel therapies for VVS, but also further establishes the sGVS rodent model as a close experimental mimetic of VVS in humans, in not only cardio- and cerebro-vascular effects, but also in behavioral changes.

## Supporting Information

S1 Fig Maximum Changes from Baseline During sGVS for MAP, HR, and CBF. (TIF)

S1 File (PDF)
