# Supplementary material for: An Experimental Model of Vasovagal Syncope Induces Cerebral Hypoperfusion and Fainting-Like Behavior in Awake Rats
Source: PLoS One. 2016 Sep 22;11(9):e0163280. doi: 10.1371/journal.pone.0163280 (PMC5033448; doi:10.1371/journal.pone.0163280)
Supplement: S1 File — (PDF) [file pone.0163280.s002.pdf]

Online Supplement

An Experimental Model of Vasovagal Syncope Induces Cerebral Hypoperfusion and Fainting-Like Behavior in Awake Rats

Devin W. McBride,[1] Cesar Reis,[2] Ethan Frank,[4] Damon W. Klebe,[1] John H. Zhang,[1, 3] Richard Applegate II,[2] and Jiping Tang[1]

[1]Department of Physiology & Pharmacology, [2]Department of Anesthesiology, [3]Department of Neurosurgery, [4]Loma Linda University School of Medicine, Loma Linda, California, USA

Detailed Methods

All experiments were carried out in accordance to the methods and procedures approved by the Loma Linda University IACUC and Research Protection Programs, and conducted in compliance with the *NIH Guidelines for the Use of Animals in Neuroscience Research*. Sixty-six adult male Sprague-Dawley rats (280-320 g) were used in this study. Rats were housed in a humidity and temperature controlled environment with a 12-hour light-dark cycle. Rats were given ad libitum access to food and water. During all surgical procedures and methods during which rats were anesthetized, body temperature was maintained at 37 ± 0.5ºC using a heating pad controlled using a rectal probe. Four animals were used in a pre-experiment to determine the optimal stimulation parameters for lowering mean arterial pressure and heart rate, and examining the effect on cerebral blood flow. Experiment 1 investigated the effect of sinusoidal galvanic vestibular stimulation (sGVS) on cerebral blood flow (groups: Sham and sGVS (n=10/group)). Experiment 2 examined the effect of sGVS in awake, freely moving rats (groups: Sham and sGVS (n=6/group)). Experiment 3 evaluated isoflurane preconditioning as a potential therapy to prevent sGVS sequelae (groups: Sham, sGVS only, and sGVS + isoflurane preconditioning (n=10/group)). Animals within each experiment were randomly assigned to a group.

Creating the Burr Hole for Cerebral Blood Flow Monitoring

One day before sGVS, rats were anesthetized using isoflurane (4% induction, 2.5% sustained, delivered in a mixture of oxygen (0.3 L/min) and medical gas (0.7 L/min)) and placed into a rodent stereotaxic frame. The scalp was shaved and disinfected (isopropanol prep pads). A midline incision was made through the skin and connective tissue, and the periosteum was separated from the skull to expose bregma and the sagittal and coronal sutures. Using a microdrill, a burr hole (3 mm in diameter) was created with the center located 5 mm proximal to the coronal suture and 4 mm right lateral of the sagittal suture. The bone flap was gently removed without damaging the underlying dura or brain tissue. After completing the burr hole, bone wax was applied to cover the burr hole and the skin was sutured. Betadine was applied to the wound and buprenorphine was administered subcutaneously (0.01 mg/kg). The animal was allowed to recover, then returned to its home cage.

Femoral Artery Catheterization

Animals were anesthetized using isoflurane (4% induction, 2.5% sustained) and placed supine. The skin over the femoral artery was shaved and disinfected (isopropanol prep pads). An incision was made and tissue dissected to expose the femoral artery. Blood flow was momentarily halted using a suture. An incision was made in the femoral artery and a PE50 catheter was inserted and advanced 10-15 mm into the femoral artery. The catheter was connected to a transducer for measurement of blood pressure and heart rate.

Laser Doppler Probe Placement

After placement of the femoral catheter, animals were gently flipped and placed into a rodent stereotaxic frame and its head secured. The sutures on the scalp were removed, the wound reopened, and the bone wax removed, exposing the dura and brain tissue. A laser Doppler probe was placed above the exposed brain tissue and used for measurement of cerebral blood flow.

Sinusoidal Galvanic Vestibular Stimulation

sGVS was induced and the optimal frequencies were determined as previously described (1-3). Briefly, after laser Doppler probe placement, animals had two Ag/AgCl needle electrodes inserted into the skin over the mastoids, behind the auditory meati. A computer-controlled stimulator (Grass Technologies, West Warwick, RI, USA) generated sinusoidal currents binaurally. In three animals, to determine the optimal stimulation parameters for inducing mean arterial and heart rate reductions, various combinations of current amplitude and frequencies were tested: 2 and 4 mA for the amplitude with frequencies of either 0.025, 0.05, 0.1, or 0.5 Hz. These four animals were not used in any other aspect of this study. sGVS was induced in sixty-two animals, used for Experiments 1-3, with 4 mA current and 0.025 Hz frequency for three minutes. Sham animals underwent all surgical procedures (*i.e.* femoral artery catheterization, burr hole, laser Doppler probe placement, electrode placement) but the stimulator was not turned on.

Blood Pressure, Heart Rate, and Cerebral Blood Flow Monitoring

Blood pressure (mmHg) and heart rate (bpm) was continuously collected and monitored using with a transducer connected to a blood pressure analyzer (Digi-Med BPA 400a, Micro-Med, Inc., Louisville, KY, USA) and the DMSI-400 software (Micro-Med, Inc., Louisville, KY, USA). Cerebral blood flow (perfusion units) was continuously collected and monitored using a laser Doppler probe (OxyFlo probe, MNP100XP, AdInstruments Inc., Colorado Springs, CO, USA) connected to a blood flow monitor (IN191, AdInstruments Inc., Colorado Springs, CO, USA) and PowerLab (PL3504, AdInstruments Inc., Colorado Springs, CO, USA) using LabChart (LabChart Pro, AdInstruments Inc., Colorado Springs, CO, USA). Blood pressure, heart rate, and cerebral blood flow were continuously monitored before sGVS (baseline values, at least 3 minutes), during stimulation (3 minutes), and for 30 minutes post-sGVS.

Awake, Freely Moving Animals

Rats were anesthetized with isoflurane (4% induction, 2.5% sustained) for placement of the electrodes. The electrodes were placed subcutaneously with only the ends inserted near the mastoids exposed. The electrodes were connected to the stimulator. Upon completion of electrode implantation, animals were allowed to completely recover from the effects of isoflurane. Total duration of isoflurane exposure during electrode placement was not longer than 10 minutes (range: 6-10 minutes). Animals were allowed to recover from the effects of isoflurane for a minimum of 50 minutes (maximum was 70 minutes). Approximately 1 hour after implantation, sGVS was induced (4 mA at 0.25 Hz and the behavior of the rat was monitored for vasovagal-like symptoms (*i.e.* fatigue, dizziness, abnormal or spastic movements, fainting).

Isoflurane Preconditioning

A cohort of animals was selected for isoflurane preconditioning before sGVS. Animals were administered isoflurane (4% induction, 2.5% sustained) in a mixture of oxygen (0.3 L/min) and medical gas (0.7 L/min). Rats inhaled isoflurane for 90 minutes each day for 5 days. Four days after completing isoflurane preconditioning, animals were subjected to the procedure for creating the burr holes (described above). Five days after the preconditioning regimen was completed, rats were subjected to femoral artery catheterization, laser Doppler probe placement, and sGVS as described above. Blood pressure, heart rate, and cerebral blood flow were measured before,

during, and for 30 minutes post-stimulation.

Data Processing and Statistical analysis

Data collected for each time range (i.e. baseline (3 minutes), sGVS (3 minutes), 0-5 minutes post-stimulation, 5-10 minutes post-stimulation, 10-20 minutes post-stimulation, 20-30 minutes post-stimulation) was post-processed to remove artifacts. Data is presented as mean ± standard deviation (SD) of the change from baseline (%). The percent change from baseline was calculated as ((average – average of Baseline)/(average of Baseline) x 100) for each time variable and group. The maximum change from baseline was determined during the 3 minutes of stimulation and is the value which had the greatest difference from baseline. Error was propagated throughout. Data was analyzed using two-way ANOVA with Sidak's *post-hoc* test (GraphPad Prism 6, La Jolla, CA, USA). A p-value of 0.05 was considered statistically significant. A p-value of 0.1 was considered as showing a tendency towards significance.

Supplemental Results

During sGVS, the maximum change from baseline for the mean arterial pressure (MAP) was not statistically different between sham rats and rats subjected to sGVS, but rats preconditioned with isoflurane had a greater change from baseline for MAP than that of sham animals ($p<0.05$) (Supplemental Figure 1). The maximum change in heart rate (HR) was significantly different for sham animals compared to that of sGVS animals ($p<0.05$) and sGVS animals preconditioned with isoflurane ($p<0.05$). Similarly, the maximum change in cerebral blood flow (CBF) was statistically different for sham rats compared to that of rats subjected to sGVS ($p<0.05$) and sGVS rats which received isoflurane preconditioning (PC) ($p<0.05$). The maximum change from baseline for all three physiological measures was indistinguishable for non-conditioned sGVS rats and isoflurane preconditioned sGVS rats.

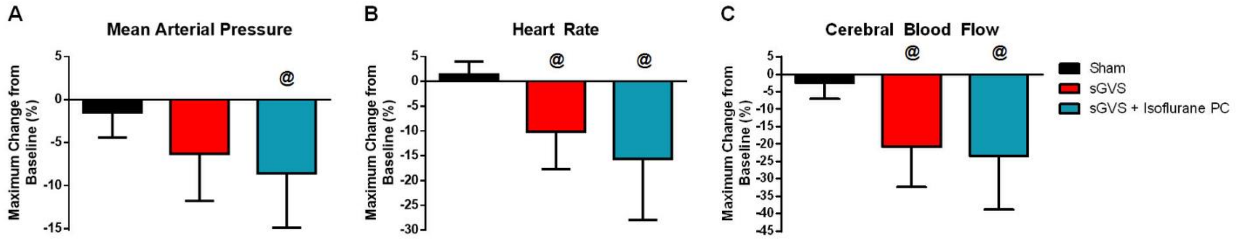

Supplemental Figure 1. Maximum Changes from Baseline During sGVS for MAP, HR, and CBF. During sGVS the maximum change for MAP was significantly greater for isoflurane preconditioned rats (sGVS + Isoflurane PC) compared to sham rats. The maximum changes in HR and CBF during sGVS was significantly greater for both the sGVS only group and sGVS + Isoflurane PC group compared Sham. @ $p<0.05$ vs Sham.

Supplemental Discussion

In the awake animal model of sGVS, approximately 1 hour was given for animals to recover from the isoflurane effects on the cardiovascular system. The total duration for isoflurane exposure before sGVS was not more than 10 minutes. Given a 10-minute exposure time, based on the study by Bailey, 90% of the isoflurane is removed from the animal by 30 minutes after stopping inhalation (11). Furthermore, based on the half-life of isoflurane (less than 5 minutes), more than 99% of the isoflurane should be eliminated by 50 minutes after halting inhalation.

Other Study Limitations

First, the choice of anesthetic is critical. Inhaled anesthetics have known cardio- and cerebro-vascular effects (4). Isoflurane causes decreases in blood pressure (5), cardiac output (6), and CBF (4), but increases HR (5, 6). While, sevoflurane, which reduces blood pressure, does not affect HR(7) or CBF.(8) Injectable anesthetics also have cardio- and cerebro-vasculature effects: reduced blood pressure, HR, and CBF (9, 10). Herein, isoflurane was chosen based on its wide use and its well-documented cardio- and cerebro-vascular effects (4-6). A second limitation of this study was the selection of the preconditioning regimen: daily 90-minute 2.5% isoflurane conditioning for 5 days. This was arbitrarily selected, and thus may not be the optimal preconditioning regimen to prevent sGVS-induced cerebrovascular depression.

References

1. Cohen B, Martinelli GP, Ogorodnikov D, Xiang Y, Raphan T, Holstein GR, et al. Sinusoidal galvanic vestibular stimulation (sGVS) induces a vasovagal response in the rat. Experimental brain research. 2011;210(1):45-55.
2. Holstein GR, Friedrich VL, Jr., Martinelli GP, Ogorodnikov D, Yakushin SB, Cohen B. Fos expression in neurons of the rat vestibulo-autonomic pathway activated by sinusoidal galvanic vestibular stimulation. Frontiers in neurology. 2012;3:4.
3. Yakushin SB, Martinelli GP, Raphan T, Xiang Y, Holstein GR, Cohen B. Vasovagal oscillations and vasovagal responses produced by the vestibulo-sympathetic reflex in the rat. Frontiers in neurology. 2014;5:37.
4. Strebel S, Lam AM, Matta B, Mayberg TS, Aaslid R, Newell DW. Dynamic and static cerebral autoregulation during isoflurane, desflurane, and propofol anesthesia. Anesthesiology. 1995;83:66-76.
5. Constantinides C, Mean R, Janssen BJ. Effects of isoflurane anesthesia on the cardiovascular function of the C57BL/6 mouse. ILAR journal / National Research Council, Institute of Laboratory Animal Resources. 2011;52(3):e21-31.
6. Perouansky M, Pearce R, Hemmings H. Chapter 20 - Inhaled anesthetics: mechanisms of action. In: Miller RD, Eriksson LI, Fleischer LA, Wiener-Kronish JP, Young WL, editors. Miller's Anesthesia. New York: Churchill Livingstone Elsevier; 2010.
7. Kanaya N, Hirata N, Kurosawa S, Nakayama M, Namiki A. Differential effects of propofol and sevoflurane on heart rate variability. Anesthesiology. 2003;98(1):34-40.
8. Shan J, Sun L, Wang D, Li X. Comparison of the neuroprotective effects and recovery profiles of isoflurane, sevoflurane and desflurane as neurosurgical pre-conditioning on ischemia/reperfusion cerebral injury. International journal of clinical and experimental pathology. 2015;8(2):2001-9.
9. Albrecht M, Henke J, Tacke S, Markert M, Guth B. Effects of isoflurane, ketamine-xylazine and a combination of medetomidine, midazolam and fentanyl on physiological variable continously measured by telemetry in Wistar rats. BMC Veterinary Research. 2014;10:198.
10. Lei H, Grinberg O, Nwaigwe CI, Hou HG, Williams H, Swartz HM, et al. The effects of ketamine-xylazine anesthesia on cerebral blood flow and oxygenation observed using nuclear magnetic resonance perfusion imaging and electron paramagnetic resonance oximetry. Brain research. 2001;913(2):174-9.
11. Bailey J. Context-sensitive half-times and other decrement times of inhaled anesthetics. Anesthesia & Analgesia. 1997;85:681-6.
